# Suppression of circulating IgD+CD27+ memory B cells in infants living in a malaria-endemic region of Kenya

**DOI:** 10.1186/1475-2875-10-362

**Published:** 2011-12-13

**Authors:** Amolo S Asito, Erwan Piriou, Walter GZO Jura, Collins Ouma, Peter S Odada, Sidney Ogola, Nancy Fiore, Rosemary Rochford

**Affiliations:** 1 Maseno University, Maseno, Kenya; 2 Center for Global Health Research, Kenya Medical Research Institute, Kisumu, Kenya; 3 SUNY Upstate Medical University, Syracuse, NY, USA; 4 Médecins Sans Frontières-Operational Centre Amsterdam, Amsterdam, The Netherlands

**Keywords:** B cells, Infant immunity, *Plasmodium falciparum*

## Abstract

**Background:**

*Plasmodium falciparum *infection leads to alterations in B cell subset distribution. During infancy, development of peripheral B cell subsets is also occurring. However, it is unknown if infants living a malaria endemic region have alterations in B cell subsets that is independent of an age effect.

**Methods:**

To evaluate the impact of exposure to *P. falciparum *on B cell development in infants, flow cytometry was used to analyse the distribution and phenotypic characteristic of B cell subsets in infant cohorts prospectively followed at 12, 18 and 24 months from two geographically proximate regions in western Kenya with divergent malaria exposure i.e. Kisumu (malaria-endemic, n = 24) and Nandi (unstable malaria transmission, n = 21).

**Results:**

There was significantly higher frequency and absolute cell numbers of CD19+ B cells in Kisumu relative to Nandi at 12(*p *= 0.0440), 18(*p *= 0.0210) and 24 months (*p *= 0.0493). No differences were observed between the infants from the two sites in frequencies of naïve B cells (IgD+CD27-) or classical memory B cells (IgD-CD27+). However, immature transitional B cells (CD19+CD10+CD34-) were higher in Kisumu relative to Nandi at all three ages. In contrast, the levels of non-class switched memory B cells (CD19+IgD+CD27+) were significantly lower overall in Kisumu relative to Nandi at significantly at 12 (*p *= 0.0144), 18 (*p *= 0.0013) and 24 months (*p *= 0.0129).

**Conclusions:**

These data suggest that infants living in malaria endemic regions have altered B cell subset distribution. Further studies are needed to understand the functional significance of these changes and long-term impact on ability of these infants to develop antibody responses to *P. falciparum *and heterologous infections.

## Background

Development of immunity is dependent on both exposures to pathogens as well as age of the host. Children living in malaria endemic regions of sub-Saharan Africa have the burden of both early age of exposure and repeated exposure to malaria while their immune system is developing. That this is problematic is evidenced by the fact that not only do children under 5 years of age suffer the highest morbidity and mortality due to *Plasmodium falciparum *infection, they also have the highest all-cause mortality of any age group living in malaria endemic regions. Several reasons have been proposed, however, it is generally agreed that this phenomenon is likely due to inefficient innate and adaptive immune responses and/or immunopathology that ensue due to disease [1-3].

During childhood, there are a number of changes in the lymphocyte compartment and these are especially evident in the period from birth through 2 years of age. Infants have significantly higher numbers of peripheral CD19+ B cells as compared to adults. And while development of germinal centres and memory B cells can occur soon after birth, the relative percentage of memory B cell expands over time and reflects the infants' antigenic history. Of note as well, is the inability of infants to respond to T independent antigens until ~ 2 years of age. Marginal zone B cells in infants express the enzyme activation induced deaminase (AID) essential for somatic hypermutation but in adults, these same cell types do not express AID [4]. The peripheral equivalent of the marginal zone cell is the IgM+IgD+CD27+CD19+ non-class switched memory B cell. These cells have been shown to have a diversity of immunoglobulin receptors with evidence of somatic hypermutation but are thought to be independent of germinal center passage [5]. This cell type increases from infancy and reaches adults values by 2-3 years of age where it composes approximated 5-10% of the total B cell compartment similar to the percentages observed for classical memory B cells (IgM-IgD-CD27+CD19+)[4]. Interestingly, splenic non-class switched IgD+CD27+ B cells are thought to be essential for rapid mobilization to blood borne pathogens as well as Streptococcus pneumonia [6]. The rapid mobilization is more typical of innate immune response than adaptive immunity and thought to emerge from TLR9 signalling of transitional B cells [7].

Chronic infections such as HIV and hepatitis C virus have been shown to perturb the distribution of peripheral B cell subsets. While *P. falciparum *is not a chronic infection per se, in infants, repeated exposures and delay of clearance of the pathogen is likely to make the host respond to *P. falciparum *more like a chronic infection. This is evidenced by the similarities in altered B cell subpopulations observed during malaria and HIV infections. For example, in both HIV and *P. falciparum *infected hosts, increases in transitional CD19+CD10+ B cells [8,9], decrease in IgD-CD27+ memory B cells [9-12], and increases in CD21^lo ^atypical exhausted B cells [8,11] have been reported. Children infected with HIV were found to have a selective depletion of non-class-switched (IgD+CD27+) memory B cells relative to healthy children [13]. A recent study in a mouse model of Plasmodium showed selective depletion of marginal zone B cells during acute *Plasmodium chabaudi *infection [14], but it is unknown if this also occurs in humans.

Given that memory B cell lineages are established during infancy and *P. falciparum *infections perturb memory B cells populations critical in the elaboration of effective humoral immune responses [9,11], there is a need for a better understanding of the effect of early exposure to *P. falciparum *infection on infant B cell development. In this study, peripheral B cell subsets were characterized in a longitudinal cohort of infants followed through 2 years of age. Infants were enrolled from two regions of Western Kenya that experience different patterns of malaria transmission intensity throughout the year, thus allowing us to ask how increasing *P. falciparum *transmission affected development of B cell compartments independent of age-related changes in the B cell compartment that occur through this period.

## Methods

### Study site and population

The study was performed using the infrastructure of two sub-district hospitals that serve rural populations: Chulaimbo sub-district hospital, in Kisumu county (a malaria holoendemic region), where residents receive 100-300 infective mosquito bites per annum and *P. falciparum *accounts for 97% of the malarial infections primarily in infants [2,15], and Mosoriot sub-district hospital in Nandi county, an area that experiences unstable malaria transmission with an estimated entomological inoculation rate of 12 infective mosquito bites per annum [16,17].

The samples used for this study were from a larger study [18] and only those participants with enough remaining peripheral blood mononuclear cells (PBMCs) for B cell immunophenotyping were included in this study. Approval of this study was obtained from both the Kenya Medical Research Institute and SUNY Upstate Medical University Ethical Review Boards. Written informed consent was obtained from the parents or guardians of study participants before any sample collection. The enrolment criteria involved recruiting pregnant women who were HIV-negative from the two study sites and following their children from 1 month of age up through 24 months. All infants were enrolled in a three-month period, from April 2006-June 2006. As part of the study participant's health care, the infants were closely monitored for any illness and all cases were reported to the study clinicians for treatment as per Kenya Ministry of Health (MOH) guidelines. A total of 38 children from Chulaimbo sub-district hospital in Kisumu county (herein referred to as Kisumu) and 36 from Mosoriot sub-district hospital in Nandi county (herein referred to as Nandi) had PBMC collected at 12, 18 and 24 months for B cell immunophenotyping analysis. At the end of 24 months follow-up there were 24 infants from Kisumu and 21 from Nandi who were captured during the three time points and all subsequent analyses were based on these sample sizes.

### Blood collection and processing

Finger-prick blood was collected in EDTA tubes and measurements of haemoglobin (Hb) levels were determined using a portable β-haemoglobin photometer (Hemocue AB Angelholm, Sweden), white complete blood counts were performed with a Beckman Coulter AcT diff2 (Beckman-Coulter Corporation, Miami, FL, USA). In addition, 1-3 ml of venous blood from the study participants into heparinized vacutainer tubes. Samples were collected at the same period of time for both sites and in any given week when infants were 12, 18 and 24 months of age, samples from infants of the appropriate age from both Kisumu and Nandi were collected and analysed. Ficoll density gradient centrifugation of blood was done within 1 h of blood collection; the plasma was removed for subsequent serologic analysis. PBMCs were analyzed immediately for immunophenotype analysis.

### Quantitative PCR investigation of *P. falciparum *parasites

Due to the fact that microscopy has been shown to underestimate the frequency of *P. falciparum *infection in asymptomatic individuals from areas with unstable malaria transmission [19], both thin/thick smear Giemsa staining as well as Q-PCR on DNA extracted from peripheral blood by quantitative PCR was performed as described [18].

### Peripheral blood mononuclear cell isolation and cell surface phenotyping

The frequencies of B cell sub-populations in PBMCs were analysed by flow cytometry and data acquired using FACScalibur (Becton Dickinson Immunocytometry Systems, San Jose, USA). The following monoclonal antibody (mAb) panels were used: pan-lymphocyte panel (anti-CD45-APC, anti-CD19-PE, anti-CD3-FITC), T cell panel (anti-CD3-APC, anti-CD8-FITC, anti-CD4-PE), B cell subset panel (anti-IgD-FITC, anti-CD27-PE, anti-CD19-APC) and transitional B cell panel (anti-IgD-FITC, anti0CD10-PE, anti-CD19-APC). Isotypic controls were IgG_2a_, K -FITC (mouse), IgG_1_, K-PE (mouse) and IgG_1_, K-FITC (mouse) (BD Pharmingen, San Diego, CA, USA). For the B cell subset analysis, a minimum of 10,000 gated CD19^+ ^lymphocytes were acquired; for the pan-lymphocyte and T cell subsets, a minimum of 10,000 lymphocytes based on FSC/SSC were acquired. Absolute cell counts for lymphocyte subsets were based on white blood cell counts obtained on each sample. All flow cytometry data were processed using FlowJo software (Tree Star Inc., San Carlos, CA, USA).

### Statistical methods

GraphPad Prism version 5 (GraphPad Software, Inc, La Jolla, CA, USA) was used for statistical analysis. Statistical difference in categorical variables between the two defined groups was determined using Fischer exact test while Mann-Whitney U test was used to determine differences in continuous variables. Age-related change in the lymphocyte populations and Ig levels were evaluated using Friedman test with post-hoc Dunns test for multiple comparisons. Correlation was done using non-parametric Spearman analysis. doing *P *values of ≤0.05 were considered statistically significant.

## Results

### Demographic and clinical characteristics of the study population

To follow infants with divergent underlying exposure to malaria, we recruited infants from two rural study sites in Western Kenya. The first site was in Nyanza Province, Kisumu District. This area experiences endemic malaria and is characterized by intense, perennial transmission [20]. The second study site was in the highlands of Rift Valley Province, Nandi District, where malaria transmission is unstable and associated with periodic outbreaks of malaria morbidity [20]. Infants were enrolled at 1 month of age, and blood samples were collected monthly through the first year and then every 3 months through two years. Venous blood draws were done at 12, 18 and 24 months. To confirm previously reported differences in malaria exposure between these two sites [20,21], the DNA samples from each cohort were analysed for *P. falciparum *DNA by Q-PCR. A significantly higher number of children with asymptomatic *P. falciparum *parasitaemia was found in the Kisumu children at any given time and higher malaria parasitaemia for children from Kisumu relative to Nandi [18]. At the time of venous blood draws, none of the infants were positive for *P. falciparum *by blood smear and all had temperatures within the normal range (Table 1). However, *P. falciparum *DNA was detected in both Kisumu and Nandi infants using the more sensitive Q-PCR method [22]. The proportions of infants that were positive for *P. falciparum *by Q-PCR were significantly higher in Kisumu relative to Nandi at 12 (*p *= 0.0331) and 24 months (*p *= 0.0025), while they were comparable at 18 months (*p *= 0.7351). In addition, age-related analysis revealed significantly higher parasitaemic infants at 12 months relative 18 and 24 months in both cohorts (both, *p *< 0.0001).

**Table 1:** Demographic, parasitological and hematological characteristics of the study population

	Kisumu				Nandi			
		
Parameter	12 months	18 months	24 months	*p-*value	12 months	18 months	24 months	*p*-value
No. of participants	24	24	24		21	21	21	
Gender								
Male (n[%])	14(58)	14 (58)	14 (58)		9 (43)	9 (43)	9 (43)	
*P. falciparum *BS+	0	0	0		0	0	0	
*P.falciparum *Q-PCR+ (n[%])*	13 (54)	5 (21)	8 (33)	**<0.0001^a^**	8 (38)	5 (24)	3 (14)	**<0.0001^a^**
Temperature °C	36.55(0.008)	36.51(0.48)	36.27(0.12)		36.64((0.34)	36.58(0.4)	36.74(0.1)	
Erythrocyte indices								
Hb conc (g/dl)	10.85(0.25)	11.07(0.26)	11.50(0.26)	**0.0390^b^**	11.21(0.26)	11.37(0.28)	11.76(0.34)	0.0524^b^
RBCs (10^6^/µl)	5.00(0.07)	4.90(0.14)	4.68(0.09)	**0.0191^b^**	4.73(0.01)	5.03 (0.08)	4.77(0.01)	**0.0019**^b^

Data presented as Mean (SEM) unless otherwise stated

Abbreviations: *Hb conc *hemoglobin concentration; *RBCs *red blood cells. Statistically significant *p *≤0.05 are in bold

^a^The differences in proportions over the three time points was determined using Fischer exact test

^b^Analysis of the continuous variables over the three time points was determined by Friedman test with post-hoc Dunns test for multiple comparisons

*Parasitemic infants were significantly higher in Kisumu relative to Nandi at 12 (*p *= 0.0331), 18 (*p *= 0.7351) and 24 months (*p *= 0.0025)

### Infants from Kisumu have elevated frequency of total CD19^+ ^B cells

**Figure 1:**
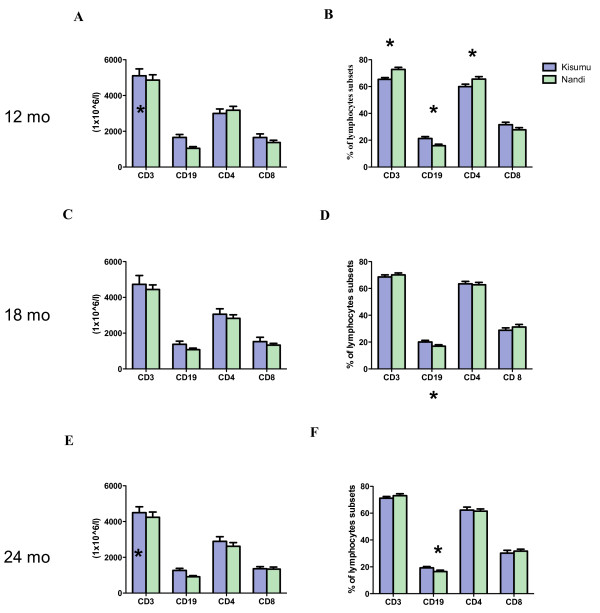
**The absolute lymphocyte counts and the frequency of lymphocyte subsets at 12, 18 and 24 months in infants from Kisumu and Nandi**. Panels **a**, **c**, and **e **show the absolute cell numbers based and panels **b**, **d**, **f **show percentages based on either CD45+CD19+ and CD45+CD3+ panel or CD3+CD4+ and CD3+CD8+ panel. * significant *P *values (<0.05). All comparison were done using unpaired t test.

To evaluate the impact of early exposure to differential *P. falciparum *transmission on infants lymphocyte subsets, freshly isolated PBMCs from Kisumu and Nandi infants were stained with anti-CD45, anti-CD3 and anti-CD19 mAB and analysed by multicolor flow cytometry to identify CD45^+^CD3^+ ^T and CD45^+^CD19^+ ^B cell subsets at 12, 18 and 24 months of age. Percentages and absolute numbers of each subset are shown in Figure 1. Infants from Kisumu had significantly higher percentages of CD19^+ ^B cells relative to Nandi at 12 (*p *= 0.0440), 18 (*p *= 0.0210) and 24 months (*p *= 0.0493). In addition, Kisumu infants also had significantly higher absolute numbers of CD19^+ ^B cells relative to Nandi infants at 12 and 24 months indicating that percentage increases in CD19^+ ^cells were not due to changes in the T cell subset. Consistent with previous reports [23,24], there was no age-related change in the frequency of CD19+ B cells in both cohorts.

To further examine T cell subsets, the percentages of CD3^+^CD4^+ ^and CD3^+^CD8^+ ^T cells was done by staining with anti-CD3, -CD4 and -CD8 mABs. Strikingly, the frequency of CD4 T cell and CD4/CD8 T cell ratio were significantly higher in Nandi relative to Kisumu at 12 months of age (*p *= 0.0294 and *p *= 0.041, respectively) (Table 2). However, no significant differences were observed at other time points. When we analysed whether there were age-dependent changes in T cell subsets, no significant differences were observed.

**Table 2:** Comparative flow cytometric analysis of peripheral lymphocytes distribution in infants from Kisumu and Nandi

	**Kisumu**				**Nandi**			
		
**Parameter**	**12 months**	**18 months**	**24 months**	***p-*value**	**12 months**	**18 months**	**24 months**	***p*-value**
		
CD45+CD19+ B	17.55	18.93	19.61	0.4233	14.80	15.81	15.77	0.8372
cells	(15.03-28.35)	(17.05-24.41)	(15.23-22.44)		(10.65-21.45)	(11.72-19.02)	(12.79-18.95)	
CD45+CD3+ T	66.35	66.94	69.62	0.0768	73.80	74.44	71.79	0.9995
cells	(59.23-70.35)	(62.42-73.87)	(66.15-75.10)		(67.70-79.45)	(68.56-76.79)	(69.47-77.74)	
CD3+CD4+ T	58.30	63.72	65.12	0.2275	70.40	62.31	61.53	0.1315
cells	(54.15-67.10)	(58.19-70.84)	(49.09-72.07)		(60.50-74.50)	(57.26-72.63)	(53.97-67.18)	
CD3+CD8+ T	32.70	25.81	26.33	0.1684	24.40	27.06	31.20	0.2144
cells	(23.85-36.75)	(21.99-34.05)	(21.58-44.75)		(19.75-31.65)	(22.06-35.69)	(25.53-33.70)	
CD4/CD8 ratio	2.18(0.27)	2.61(0.33)	2.18(0.32)	0.5280	2.83(0.27)	2.67(0.35)	2.13(0.20)	0.0571

Freshly isolated PBMCs from Kisumu (n = 24) and Nandi (n = 21) infants were analyzed by multicolor flow cytometry following staining with various combinations of monoclonal antibodies (mAB). The data are presented as median (25^th^-75^th ^percentiles) of the lymphocyte subset analyzed unless otherwise stated. Statistical differences between the two cohorts was determined by Mann-Whitney U test while age-related changes in lymphocyte subset within cohorts was analyzed using Friedman test with post-hoc Dunns test for multiple comparisons. Statistically significant *p-*values of ≤0.05 are in bold

### Expanded populations of immature transitional B cells in infants from a malaria-endemic region

**Figure 2:**
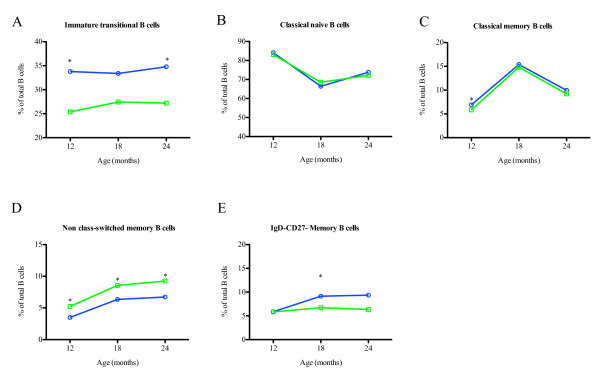
**Changes in B cell subsets in infants from Kisumu and Nandi**. Panels **a-e **are the different B cell subsets as determined by flow cytometry as described in Figure 3 and relative frequencies are shown. The different B cell subsets are expressed as a percentage of total CD19+ B cells for both Kisumu and Nandi infants. The circles and blue lines represent Kisumu infants while the squares with green lines represent Nandi infants. Statistical significance in continuous variables between the two cohorts was measured by Mann-Whitney U test while age-related change within the cohorts was measured using Friedman test with a Dunns post-hoc test for multiple comparisons. The asterisk (*) denotes significant *p*-values ≤0.05.

Expansion of the immature transitional CD10^+^CD34- B cell population is observed during acute clinical *P. falciparum *malaria, HIV infection and in autoimmune diseases [9,25]. However, these studies have only been performed in adults and children above 2 years of age. To determine if there were differences in the frequency of immature transitional cells in our cohort, PBMCs were analysed for the presence immature transitional B cells. The frequency of immature transitional B cells (CD19+CD10+CD34-) was expanded in Kisumu relative to Nandi at all three times points although only significantly higher in Kisumu at 12 (*p *= 0.0233) and 24 months of age (*p *= 0.0338). However, no significant age-related change in the frequency of this subset was observed in both cohorts (Figure 2a).

### Suppressed levels of (IgD+CD27+) non class-switched memory B cells in infants from malaria-endemic region

**Figure 3:**
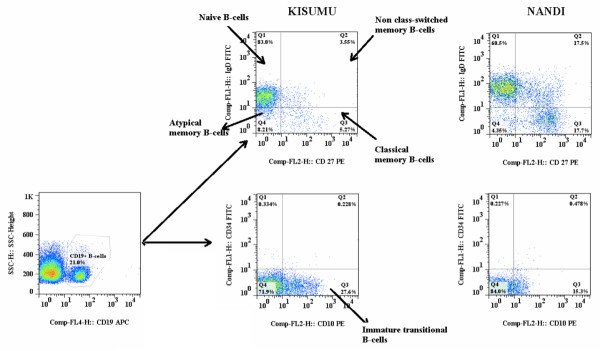
**A representative multi-color flow cytometry gating strategy to quantify different B cell subsets in Kisumu and Nandi infants**. The B cell subsets FACS plots are of a representative Kisumu and Nandi infant. Within the live CD19+ B cell gate, the B cell subsets were discriminated as follows: immature transitional B cells (CD19+CD10+CD34-), classical naive B cells (CD19+IgD+CD27-), classical memory cells (CD19+IgD-CD27+), non class-switched memory B cells (CD19+IgD+CD27+) and CD19+IgD-CD27- B cells.

Viral and bacterial infections in paediatric populations are associated with depletion of non-class-switched memory B cells [5,11,13,26]. However, whether there is diminution of this subset in infants living in areas with high intensity of *P. falciparum *transmission has not been analysed. Moreover, there are no studies that directly address age-related changes in the frequency of this population in infants. To analyse the effects of age and P. falciparum transmission on development of B cell subsets, PBMCs were stained with CD19, IgD and CD27 mABs to discriminate naive (IgD+CD27-) from three memory B cell subsets; classical (IgD-CD27+), non class-switched (IgD+CD27+) and IgD-CD27-. This latter subset is a heterogeneous subset that contains both transitional (IgD-CD10+) and atypical memory (IgD-CD27-) B cells (Figure 3). Because only three-color flow cytometry analysis was available, it was not possible to further distinguish between these two populations.

As expected, naive CD19+IgD+CD27- B cells were the predominant population in both cohorts and at all time points (Figure. 2b). The percentages decreased over time. Concomitantly, significant age-related increases in the frequency of in classical memory CD19+IgD-CD27 in both cohorts (*p *< 0.0001) were observed (Figure 2c). With the exception of a slightly but significantly higher percentage of CD19+IgD-CD27+ B cells at 12 months of age in the Kisumu infants relative to the Nandi infants (*p *= 0.049), no other differences between the cohorts were noted. Multiple comparisons test revealed a significant age-related change in the frequency of this subset in both cohorts (*p *< 0.0001). Interestingly, when comparison was made in the percentage of cells that were IgD+CD27+, a reduced population of IgD^+^CD27^+ ^B cells was observed in Kisumu relative to Nandi at 12, 18 and 24 months (*p *= 0.0144, *p *= 0.0013 and *p *= 0.0129, respectively) (Figure 2d). Moreover, there was a significant age-related increase in the proportion of this subset in both cohorts (both, *p *< 0.0001). These data also revealed elevated frequency of CD19+IgD-CD27- B in Kisumu at 18 months (*p *= 0.0278) relative to Nandi. While multiple comparison analysis revealed no significant age-related change in the Nandi cohort (*p *= 0.6618), there was a significant increase in Kisumu cohort over time (*p *< 0.0008), with post-hoc analysis revealing lower percentages at 12 months relative to 18 and 24 months (all, *p *< 0.05, after a Dunns test for multiple comparisons) (Figure 2e). A summary of absolute B cell counts and the differences among the groups analysed is also shown in Additional file 1.

While none of the infants in the study at the time of sample collection had evidence of clinical malaria which would be indicated by fever greater than 37.5°C and detectable parasites on peripheral blood smear greater than 5000 (all were blood smear negative), there were infants that were *P. falciparum *positive based on detection of parasite DNA by Q-PCR. To determine if there was an impact of being *P. falciparum *DNA positive on different frequencies of CD19+ B cells, IgD+CD27+ B cell subset and the CD10+CD34- B cell subset, we did a Spearman correlation and found that being *P. falciparum *DNA positive did not correlate with increased or decreased frequencies of IgD+CD27+ B cell subset and the CD10+CD34- B cells.

## Discussion

Impaired humoral immune protection associated with prenatal or chronic exposure to *P. falciparum *is a common immunological abnormality in paediatric populations from malaria-endemic regions [2,27]. Alterations in B-cell homeostasis [9,11], and longevity, quantity and quality of humoral responses based on age and malaria transmission dynamics have previously been reported in children [2,3,27]. However, it is unknown what the impact of early differential exposure to *P. falciparum *on infant B cell development. In this study, the development of B cell subsets was investigated in infants followed through 24 months of age from geographically proximate regions of Kenya that experienced divergent *P. falciparum *transmission dynamics. Infants from the malaria-endemic area had higher overall numbers of CD19+ B cells but reduced populations of circulating IgD+CD27+ memory B cells and expanded populations of CD10+CD34- immature transitional B cells at all ages relative to infants living in an area with unstable malaria transmission. These data suggest that early exposure to *P. falciparum *infection results in dysregulation of the development of B cell subsets in infants.

Increased frequency and absolute numbers of CD19+ B cells in infants from malaria-endemic region relative to those from an area with unstable malaria transmission at all ages examined suggests that early age or even prenatal exposure to *P. falciparum *infection results in the expansion of the B cell compartment. The expansion of the immature transitional B cells in the peripheral blood observed in this study as well as previously reported during acute clinical malaria in infants [9], could result in increases in the total CD19+ population. Moreover, the *P. falciparum *erythrocyte membrane proteins 1 (PfEMP1) is a polyclonal B cell activator [28] and could potentially drive expansion of B cells. Overall, these data suggest that impaired antibody responses common in infants from malaria-endemic regions is not due to decline in the frequency of total CD19+ B cells.

Previous studies of individuals with HIV [25], acute clinical malaria [9] systemic lupus erythematosus [29] and X-linked lymphoproliferative disease [30] have found an expansion of immature transitional B cells characterized by expression of CD10. These cells lack CD34 expression [9] and have expression of IL-7 [25] as well as CD24 and CD38 [29,30]. In this study, there was an expansion of this sub-population of immature transitional B cells in infants from Kisumu relative to Nandi at all ages examined. Expansion of transitional B cell populations correlated with impaired humoral immunity [30] thus suggesting the expansion of this population in infants from a malaria-endemic area could contribute to impaired humoral immunity. However, it should be noted that consistent with previous studies, both cohorts of infants had higher levels of transitional B cells than observed in adult counterparts [30].

There is some controversy over whether CD19+IgD+CD27+ B cells in peripheral circulation represent circulating marginal zone B cells[4] or non-class switched memory B cells [31]. It is clear however that this B cell subset is present in the peripheral blood of infants and increases with age [5] and loss of CD19+IgD+CD27+ B cells is observed in pediatric HIV infection [13]. A decline in the frequency of IgD+CD27+ memory B cells was observed in circulation of infants from malaria-endemic regions relative to those from an area with unstable malaria transmission at all ages. Although the immunological pathways that orchestrate the depletion of this subset in infants from malaria-endemic region is unknown, splenectomy has been associated with reduced peripheral circulation of this subset in autoimmune patients [32]. Interestingly, *P. falciparum *infection has been shown to disrupt splenic architecture [33], and thus, may interfere with peripheral homeostasis of this B cell subset. Early exposure to *P. falciparum *may interfere with splenic development and/or disrupt splenic architecture in infants from malaria-endemic region, which could be one possible explanation for the diminution of CD19+IgD+CD27+ B cells observed in the cohort of infants from Kisumu. Moreover, since this subset produce poly-reactive IgM important in innate immunity against bacterial and viral pathogens[4], the early depletion of this population may result in increased susceptibility to bacterial or viral infections, and reduced efficacy of pneumococcal vaccines in infants from malaria-endemic regions [34,35]. The age-related increase in the frequency of this subset in both cohorts observed is consistent with previous studies showing an age-dependent increase in this B cell subset in infants [4].

There were several limitations of this study. First, since infants were enrolled over a three-month period of time changes in malaria transmission could have occurred. However, we analysed samples from both sites every week over the three-month period to minimize any differences that time of year would have on the results. In addition, while there are well-documented differences in malaria transmission between Kisumu and Nandi, it was not possible to generate an accurate record of the number of malaria infections in the infants in our cohort over the period of observation. In rural areas in Kisumu, infants can be treated at home with anti-malarial drugs bought from local shops. Home treatment could reduce measurable parasitaemia. Because only a monthly follow-up was available in the first year, some episodes of malaria could be missed. In addition, the instability of antibody responses to malaria [3] in infancy prevented using detection of malaria antibody as a surrogate for malaria infections. Finally, it would be ideal to have a more elaborate B cell phenotyping panel, but at the time of this study, only a three-colour analytic flow cytometer was available for analysis in Kenya. For example, while an elevated frequency of CD19^+^IgD-CD27- B cells was noted in infants from Kisumu relative to Nandi, because this population includes both immature transitional B cell subset (IgD-CD27-CD10+) as well as the atypical exhausted memory cells, the significance of this observation cannot be determined.

## Conclusions

In conclusion, these results demonstrated that there were profound differences in B cell subsets in infants from geographically proximate regions with divergent malaria exposure that were independent of the maturation of the immune system in infants. These data further provide evidence that impaired humoral immune response in infants living in malaria endemic regions may be a result of multifactorial factors, including suppression of IgD+CD27+ memory B cells, and expansion of immature transitional B cells. Future work is needed to elucidate the effects of early expansion of immature transitional B cells and depletion of IgD+CD27+ memory B cells on infants functional immunity and whether malaria control programmes targeting pregnant women and their infants can preserve B cell development in infants from malaria-endemic regions.

## Competing interests

The authors do not have any commercial or other association that might pose a conflict of interest.

## Authors' contributions

ASA participated in study design, carried out the cell isolation, cell analysis and drafted the manuscript. ER participated in the design and coordination of the study and draft of manuscript. PSO, SO and NF participated in recruitment, FCF, ELISA and PCR analysis. WGZOJ and CO carried out the statistical analysis and drafted the manuscript. RR conceived of the study, and participated in its design and coordination and drafted the manuscript. All authors read and approved the final manuscript.

## Supplementary Material

Additional file 1 **Absolute lymphocyte counts for the different B cell subpopulations in infants from areas with divergent malaria exposure according to age**. Click here for file
