# Supplementary material for: Suppression of circulating IgD+CD27+ memory B cells in infants living in a malaria-endemic region of Kenya
Source: Malar J. 2011 Dec 13;10:362. doi: 10.1186/1475-2875-10-362 (PMC3315680; doi:10.1186/1475-2875-10-362)
Supplement: Additional file 1 — Absolute lymphocyte counts for the different B cell subpopulations in infants from areas with divergent malaria exposure according to age. [file 1475-2875-10-362-S1.docx]

| Additional file 1. Absolute lymphocyte counts for the different B cell subpopulations in infants from areas with divergent malaria exposure according to age. | | | | | | | | |
| --- | --- | --- | --- | --- | --- | --- | --- | --- |
|  | Median absolute B cell subset counts (25th and 75th percentiles) for the indicated age group | | | | | | |  |
|  | Chulaimbo | | | *p*-value | Mosoriot | | | *p* value |
| Subset | 12(n=24) | 18(n=24) | 24(n=24) |  | 12(n=21) | 18(n=21) | 24(n=21) |  |
| CD19+ total B cells | 1942  (1384-3718) | 2252  (1377-2989) | 2253  (1307-2579) | 0.7990 | 1428  (1012-2293) | 1350  (1158-2213) | 1565  (1184-1752) | 0.9945 |
| CD19+IgD+CD27- | 1614  (1157-3050) | 1436  (895-1830) | 1592  (817-1956) | 0.2866 | 1195  (754-1779) | 972  (792-1412) | 1100  (958-1296) | 0.7197 |
| CD19+IgD+CD27+ | 79  (34-134) | 118  (83-161) | 142  (95-181) | **0.0092** | 78  (36-149) | 132  (96-185) | 159  (113-230) | **0.0067** |
| CD19+IgD-CD27- | 117  (70-171) | 182  (84-336) | 173  (88-261) | 0.0720 | 77  (54-126) | 102  (69-156) | 90  (51-182) | 0.4419 |
| CD19+IgD-CD27+ | 142  (88-260) | 286  (164-498) | 201  (123-293) | **0.0199** | 94  (51-121) | 232  (144-321) | 132  (102-204) | **0.0002** |
| CD19+CD34-CD10+ | 753  (509-998) | 699  (473-967) | 767  (432-1019) | 0.9476 | 415  (204-653) | 359  (234-669) | 453  (302-570) | 0.8100 |
| Friedman test was used for age-wise comparisons and *p<0.05* was considered significant. Statistically significant values are in bold | | | | | | | | |
